# Research on Labor Market Institutional Environment and Labor Agglomeration—Analysis Based on Dynamic Spatial Panel Data

**DOI:** 10.3389/fsoc.2021.738134

**Published:** 2021-10-08

**Authors:** Huaping Guan, Jianwu Zhang, Xiaoping Zhu

**Affiliations:** ^1^ School of Economics and Trade, Guangdong University of Foreign Studies, Guangzhou, China; ^2^ Zhongluotan Education and Guidance Center, Guangzhou, China

**Keywords:** labor agglomeration, labor dispute, spatial durbin model, new economic geography theory, professional and technical talents

## Abstract

The institutional environment not only has a great impact on economic development but also has a certain impact on labor agglomeration. This paper explains the phenomenon of labor agglomeration from the perspective of the labor market institutional environment. Taking the labor market institutional environment including social contract environment and labor contract environment as the premise, it is considered that a good labor institutional market can promote labor agglomeration and technological progress can stimulate the agglomeration of highly-skilled talents through theoretical analysis. Based on the provincial panel data from 2001 to 2017, the empirical analysis is conducted to verify the relevant factors through the dynamic spatial Durbin model. The paper finally puts forward some policy suggestions to maintain a good institutional environment of the labor market, including that the local government should continue to improve the labor dispute settlement system, strengthen the protection of labor rights and interests, and enhance the institutional environment of the regional labor market.

## Introduction

Since the reform and opening-up, great changes have taken place in China’s regional economic pattern. The eastern region has taken the lead in economic development because of its earlier acceptance of international industrial transfer and integration into the international production and trade system. It leads to the migration of the labor force from the central and western regions to the eastern region and from rural to urban areas. In recent years, China’s economic agglomeration and labor agglomeration pattern have undergone profound changes. On one hand, the rural surplus labor is exhausted, which gradually weakens the trend of labor agglomeration from rural to urban areas. Cai Fang (2011) (Cai and Yang, 2011) believed that the rural surplus labor force had dried up on the whole, and the Lewis turning point of labor transfer had arrived. The data from the floating population survey of the National Health and Family Planning Commission shows that the proportion of the floating population choosing to work nearby has gradually increased. Specifically, the total floating population in China showed a steady downward trend from 2015 to 2017: in 2015, the total floating population was 247 million, down by about 6 million from 2014; in 2016, the scale of the floating population reduced by 1.71 million compared with 2015, and it continued to decrease by 0.82 million in 2017. On the other hand, the rapid economic development of central cities in the central and western regions has attracted a large population and labor force to gather in these cities such as Chengdu and Xi’an. It also leads to the trend of unidirectional labor mobility from the central and western regions to the eastern regions shifting into a bidirectional flow trend. In the case of the relatively flat trend of labor migration from rural to urban areas, labor migration in different cities (regions) has become the mainstream. Therefore, it is a very meaningful topic to study the motivation of labor agglomeration in different regions (cities) of China.

**FIGURE 1:**
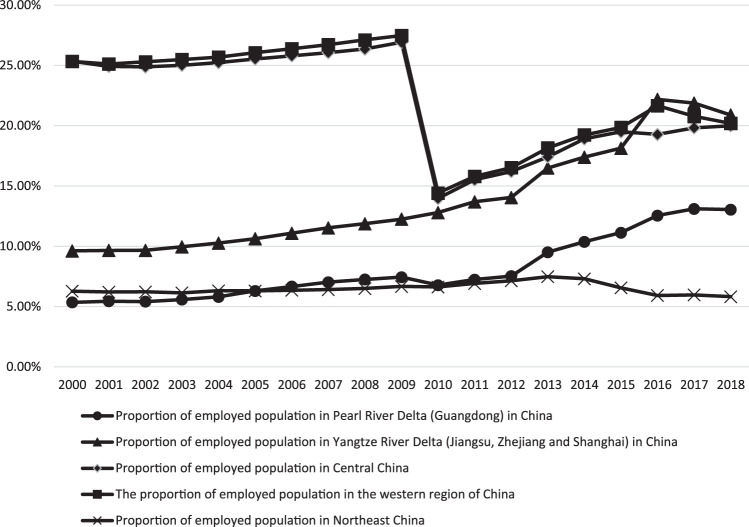
proportion of employed population in China by region, 2000–2018.

As we can be seen from Figure 1, from 2000 to 2016, the proportion of employment in the Pearl River Delta and the Yangtze River Delta gradually increased, but there were signs of a reversal of this trend after 2016; the proportion of Northeast China has shown a general downward trend since 2000; the proportion of employment in the central and western regions has gradually increased since 2010. It can be seen from these phenomena that China’s labor force presents a trend of agglomeration to some extent.

Data source: China Labor Statistics Yearbook (2001–2019).

Labor agglomeration always complements economic agglomeration. According to the new economic geography theory, the main reason for the sustained economic agglomeration is the increasing returns to scale (Krugman, 1991) (Paul, 1991) and the main reason for maintaining the increasing returns to scale is the relative monopoly income brought by technological progress and knowledge spillover (Chen Qiangyuan, 2014) (Chen and Qi, 2014). Certainly, scholars have been concerned about the impact of the institutional system on economic development. Acemoglu (2001) (Acemoglu et al., 2001) proposed that the institutional system determines the economic performance of a country (region). There are significant differences in the institutional system between countries (regions) for various reasons including history, culture and natural endowment, which has a significant impact on economic development. From the perspective of the labor market, the government’s effective regulation of the labor market can reduce the uncertainty and promote the efficiency of the labor contract (Bathelt, 2014) (Bathelt and Glückler, 2014)^.^ It is a feasible perspective to study the phenomenon of economic agglomeration from the aspect of the institutional environment. The institutional environment in different regions of a country is generally at the same level, but there are still some delicate differences. Such differences also have a certain impact on long-term economic performance. The measurement of institutional environment can be measured as a whole or partially. Thus, this paper attempts to analyze the effects of the labor market institutional environment on labor agglomeration.

The institutional environment of the labor market includes the labor contract environment between workers and enterprises (internal market environment) and the social contract environment between workers and local governments (external market environment). The labor contract environment is mainly embodied in labor contracts, wages and welfare while the social contract environment is mainly embodied in public services, social welfare and labor dispute settlement. These can be considered to be the main economic causes of labor mobility and agglomeration. A good labor contract environment means that the workers’ expectations of wages, benefits and working conditions could be satisfied, and a good social contract environment means that the labor force enjoys sound social welfare, public services, efficient government services and labor relations. It can be considered that the external environment of the labor market with stable expectations can reduce the transaction cost of labor supply and demand, improve the efficiency of the labor contract, and benefit the labor agglomeration.

### Review of Relevant Research Literature at Home and Abroad

The relevant research on the motivation of labor agglomeration includes the following perspectives:

First, from the perspective of economic motivation, it is considered that the basic motivation of labor mobility is the change of labor income. The labor market in developing countries is divided into two groups: urban areas and rural areas and the flow of the labor force between urban and rural areas is always a topic concerned by economists. For example, the Lewis model (1954) (Lewis, 1954) as a classical labor flow model explains that economic factors lead to the labor force flowing from rural to urban areas. When the marginal output of labor in urban areas is equal to that of rural areas, the rural surplus labor force stops flowing to urban areas and the Lewis turning point is established. A two-sector model of labor flow proposed by Harris and Todaro, (1970) shows that the flow of the labor force from rural to urban areas is due to the expected income gap between urban and rural areas, which could occur not only before Lewis turning point but also in the case of no surplus labor force. The technological progress occurring in cities expands the urban-rural income gap and has an important impact on the labor flow. It also contributes to the re-matching of the labor force which may have an agglomeration effect or dispersion effect (Peng Guohua, 2015 (Peng, 2015); Mikhail, 2011 (Martynovich and Lundquist, 2016); Simonen, 2016 (Simonen et al., 2016)). Agricultural technological progress leads to the decrease of demand for the agricultural labor force and promotes the transfer of the labor force from agricultural sectors to industrial sectors (Xiao Linzi, 2014 (Xiao and Xiao, 2014); Liang Xiangdong, 2017 (Liang and Yiyi, 2017))。Some scholars also comprehensively consider the benefits and costs in the process of labor agglomeration, analyze the multi-regional spatial equilibrium state under any spatial background, construct a spatial equilibrium model, and study the evolutionary characteristics of labor agglomeration (Cheng Jinwen, 2018) (Cheng and Yang, 2018).

Second, from the perspective of institutional environment, it is considered that a good institutional environment can promote labor agglomeration. A good institutional environment can reduce transaction costs, improve the development of contract-intensive and division-intensive industries, and promote the agglomeration of a high-quality labor force. The empirical analysis of the data verified that public services as a part of the external environment of the labor market have positive effects on promoting labor (population) agglomeration. (Tiebout, 1956 (Charles, 1956); Day, 1992 (Day, 1992); Dahlberg, 2012 (Dahlberg et al., 2012); Cheng Mingwang, 2019 (Cheng and Liu, 2019); Han Feng, 2019 (Han and Li, 2019); Ren Xiping, 2019 (Ren and Yin, 2019)). The conclusion also can be found in the studies of some Chinese scholars. Through investigation and empirical analysis, they found that providing more public services for the floating population is conducive to the labor agglomeration and stable work in cities (Xia Yiran, 2015 (Xia and Lu, 2015); Hou Huili, 2016 (Hou, 2016)). There are also some scholars studying the impact of the social security system on the transnational flow of the labor force. Doris (1998) (Geide-Stevenson, 1998) believed that the unsound social security system of a country will lead to the migration of talents and capital to countries with a sound social security system. John (2005) (Hassler et al., 2005) constructed a dynamic general equilibrium model to analyze transnational labor mobility and found that improving the standard of unemployment insurance could reduce the probability of labor mobility. Sana (2000) (Sana and Massey, 2000) analyzed the questionnaires about Mexicans in the United States and found that the well-established pension system is an important driving factor for Mexican labor to immigrate to the United States. The labor system environment also includes the labor contract environment between workers and enterprises. The incomplete contract theory (Grossman, 1986 (Grossman and Hart, 1986)) holds that a good contract environment can reduce the probability of “holdup” and other speculative activities, and promote specific investments. Li Junqing (2016) (Li and Liu, 2016) found that better contract enforcement can facilitate the optimization of industrial structures. Rafiqui (2009) (Pernilla, 2009) regarded that institutional environment can reduce uncertainty and affect economic agglomeration; Storper (2010) (Stroper, 2010) argued that institutional environment took some effects on population agglomeration and urban expansion. Roberto (2019) (Roberto and Rodríguez-Pose, 2019) used the data of enterprises in Western Europe to illustrate the effects of institutional quality on labor productivity and concluded that government efficiency was the most important institutional factor to determine the level of productivity.

From the above literature reviews, most of the literature based on national data support the positive effects of institutional environment on economic development and labor agglomeration. China has a vast geographical area. Due to culture and other factors, there are some differences in the institutional environment between provinces and between cities. This paper aims to study the impact of the institutional environment of the labor market in different regions of a country on labor agglomeration. The possible marginal contribution of this paper is to expand the research perspective of the motivation of labor agglomeration.

## Theoretical Framework

According to the traditional Cobb-Douglas production function, it is assumed that the economic output function of a region can be formulated as:

$$Y_{t}=A_{t}K_{t}^{\alpha }{\left(\theta_{t}L_{t}H_{t}\right)}^{1-\alpha }$$

$A_{t}$ is the level of technology in the a region, $K_{t}$ is the level of capital input, $L_{t}$ represents the scale of the labor force in the region, $H_{t}$ is the level of human capital in the region, and $\theta_{t}$ is the proportion of the labor force matching in the region. The equilibrium growth rate is formulated as:

$$\frac{\Delta Y}{Y}=\frac{\Delta A}{A}+\alpha \frac{\Delta K}{K}+\left(1-\alpha \right)\left(\frac{\Delta \theta }{\theta }+\frac{\Delta L}{L}+\frac{\Delta H}{H}\right)$$ (1)

From Eq. 1, we can see that the balanced growth rate depends on the capital growth rate, the technological progress rate and the comprehensive human capital growth rate in which depends on the matching rate, the labor growth rate and the human capital growth rate.

At the same time, according to the model constructed by Blanchard&Diamond (2010) (Blanchard et al., 1990), the labor market matching model is constructed as:

$$m_{t}=B_{t}{\left(L_{t}\left(1-\theta_{t}\right)\right)}^{\beta }{\left(v_{t}\right)}^{1-\beta }$$

Matching efficiency $B_{t}$ is positively correlated with the labor system environment; the number of job vacancies $v_{t}$ is related to the technical level which is formulated as $v_{t}=\varphi A_{t}^{\varepsilon }$ ; $m_{t}$ is the number of new labor. Then the labor growth rate can be expressed as:

$$\frac{m_{t}}{L_{t}}=B_{t}{\left(1-\theta \right)}^{\beta }{\left(\frac{v_{t}}{L_{t}}\right)}^{1-\beta }$$ (2)

It can be seen from Eq. 2 that the growth rate of the labor force is closely related to the matching efficiency $B_{t}$ . Because the institutional environment can reduce the market transaction cost and improve the matching efficiency of the labor market, the labor growth rate is positively correlated with the institutional environment. Besides, the growth rate of the labor force is positively correlated with the number of job vacancies and technological progress and is inversely proportional to the overall employment scale. The conclusion can be drawn by plugging Eq. 2 into Eq. 1 as follows:

1) Technological progress is still the main driving force of economic growth. In addition to promoting the development of total-factor productivity, it can also create more job opportunities, facilitate the matching of labor and market, and spur economic growth.
2) A good institutional environment of the labor market can not only promote the matching of human capital and technological progress but also further boost economic growth.

## An Empirical Analysis of the Impact of the Labor Market Institutional Environment on Labor Agglomeration

### Quantitative Indicators of the Labor Market Institutional Environment

The supply and demand of the labor market are not only determined by the supply and demand of labor but also affected by the government regulation of the labor market. And the most important government regulation of the labor market is the coordination of labor relations. With the promulgation of the Labor Contract Law in China in 2008, the government has strengthened the regulation on labor relations. There are relevant legal provisions whatever it is about the signing of labor contracts, the purchase of social insurance, or the settlement of labor disputes. According to the reference point theory (Fehr, 2011) (Fehr et al., 2011), the government regulation on labor relations is an important reference point for employers and employees. The change of government regulation is equivalent to the change of reference point, which can affect the behavior of employers and employees. Some differences of the law enforcement scale between regions leads to some differences of the labor institution environment between regions. In addition, the level of economic development and that of public services are also quite different. These institutional environments which are directly or indirectly related to employment have significant effects on agglomeration and dispersion of the labor force.

In this paper, labor agglomeration refers to the phenomenon that labor force concentrates from different regions to a certain region or several specific regions in a certain period of time due to the relevance of employment; This concept is similar to the concept of human capital agglomeration proposed by Simon, (1998). In the part of empirical analysis, we aim to analyze the reasons for labor agglomeration from three perspectives: 1) Job opportunities in the region. Regional economic development is positively correlated with labor demand, which means that the more job opportunities there are, the more labor forces are agglomerated; 2) The degree of protection for rights and benefits related to workers in the region. It can be measured from the signing rate of the labor contract, the settlement of labor dispute, and other aspects related. Good protection of the rights and interests of workers leads to the agglomeration of labor in the region. 3) The social welfare in the region. If workers can enjoy better basic public services such as health care and education when they are employed, it will be more conducive to the agglomeration of the labor force in the region.

This paper selects the panel data of 31 provinces in mainland China (no including Hong Kong, Macao and Taiwan region) from 2001 to 2017 and sets up variables: the number of employed people (y) in each region is replaced by the number of employed people in China Labor Statistics Yearbook; the total economic volume (GDP) of each region is replaced by the gross domestic product of each region; the total annual investment (invest) is replaced by the fixed asset investment data in the Statistical Yearbook; the research and development level of each region (RD) is replaced by the number of patent applications of each region; the level of import and export (trade) is replaced by the total amount of import and export trade of each region; the level of public welfare (ss) is replaced by the public financial expenditure of each region; as for the index of labor institutions environment (institution), two different indexes are adopted that are the number of labor dispute registrations and the rate of labor dispute settlements (jal) which divides the number of closed cases by the number of labor disputes registrations in the current year. The descriptive statistics of these variables are shown in Table 1.

**TABLE 1:** Descriptive statistics of variables.

Variable name	Observed value	Mean value	Standard deviation	Minimum value	Maximum value
**lny**	527	7.484	0.91	4.839	8.82
**lngdp**	527	8.943	1.203	4.936	11.404
**lninvest**	527	8.4	1.297	4.422	10.919
**lntrade**	527	15.892	2.3	6.871	20.286
**lnss**	527	7.308	1.077	4.369	9.618
**lnrd**	527	8.666	1.834	1.946	12.715
**Injal**	527	0.987	0.048	0.73	1.268
**Instit02**	527	1.514	1.005	−0.875	4.298

### Spatial Panel Model Analysis

Since the labor force always flows in different spaces and the level of the labor force in different regions shows a certain spatial correlation, the spatial panel data model is more suitable for this study. Besides, we can use the global Moran'Ⅰ index to judge whether there is spatial correlation. Table 2 shows that the explained variables (total labor force of each province) have obvious spatial correlation. The regional institutional environment can affect the labor agglomeration in adjacent areas. The reason is that a good regional institutional environment and economic development will drive the economic development of adjacent areas and promote the agglomeration of labor force in adjacent areas. The specific mechanisms include: first, the regional coordinated development policies can drive the development of relatively underdeveloped areas, such as the integrated development strategy of the Yangtze River Delta makes Jiangsu, Zhejiang and Shanghai drive the economic development of Anhui; Second, a good institutional environment can not only promote technological innovation in the region, but also drive technological progress in adjacent areas through technology spillover, and stimulate economic development and labor agglomeration in adjacent areas.

**TABLE 2:** Moran ’I index and Test of Spatial Correlation.

Variable	I	E(I)	sd(I)	Z	*p*-value
**y**	0.231	−0.034	0.111	2.395	0.008
**jal**	−0.07	−0.034	0.106	−0.34	0.367
**ss**	0.002	−0.034	0.109	0.338	0.368
**gdp**	0.168	−0.034	0.107	1.895	0.029
**invest**	0.259	−0.034	0.109	2.688	0.004
**rd**	0.187	−0.034	0.102	2.167	0.015
**Trade**	0.133	−0.034	0.093	1.795	0.036

The key of the spatial panel data model is to determine the spatial weight matrix. In general, it can be considered that labor mobility is closely related to geographical distance and the surplus labor in a region tends to transfer to neighboring developed regions. In this paper, two spatial weight matrices are set up consisting of the spatial proximity matrix $W_{1}$ and the space distance matrix $W_{2}$ .The setting of spatial adjacency matrix is consistent with the previous literature. The weight of adjacent provinces (regions) is 1, and the weight of non-adjacent provinces is 0. The weight matrix of spatial distance takes the reciprocal of the coordinate distance of the provincial capital city as the weight. To ensure the completeness of the matrix, the weight on the diagonal of the matrix is set to 0.

w1={1dij i≠j0     i=jdij  is the distance between region i and region j

$$w_{2}=\{\begin{array}{c} 1\;\;\;\;\;i\neq j\;Region\;i\;is\;adjacent\;to\;region\;j\\ 0\;\;\;\;\;i\neq j\;Region\;i\;is\;not\;adjacent\;to\;region\;j \end{array}$$

The common spatial panel data models include spatial lag model (SLM), spatial error model (SEM), and spatial Durbin model (SDM). The main purpose of the SLM is to explain the spatial lag of the impact of other variables. The SEM focuses on the systematic influence and effects of systematic error terms (culture, climate and environment) on the model in addition to the explanatory variables. The SDM is the generalization of the above two models which has the advantages of the two models. In this paper, the stationarity of time series data should be tested because of the over 10-years time span. We used the LLC test of panel data to test the stationarity and found that the variables in the model are stable after logarithm (see Table 3 for details).

**TABLE 3:** Variable stability test.

Variable	t-statistic	*p*-value	Variable	t-statistic	*p*-value
**lny**	−4.7440	<0.001	Lninvest	−13.0580	<0.001
**Lnjal**	−11.1172	<0.001	Lnrd	−1.3665	0.0859
**Lnss**	−9.3048	<0.001	Lntrade	−10.3416	<0.001
**Lngdp**	−13.2714	<0.001	—	—	—

The paper adopted test ideas proposed by Elhorst (2014) (Elhorst, 2014) that are“from specific to generaland”“from general to specific”. The LM [robust LM] test was used to judge whether the SLM or SEM was suitable for the analysis in this study. Wald test and LR test were conducted to determine whether the SDM could be used. Hausman test was used to determine whether the fixed effect model was used. Tests are judged by the AIC value and significance of the coefficient. The relevant tests were completed in MATLAB software and the results of spatial model diagnosis are reported in Table 4.

**TABLE 4:** diagnosis results of spatial model.

Test object	Statistic	*p* Value
**Wald test of SDM vs SAR model**	53.8038	8.0807e-10 (<0.001)
**LR test of SDM vs SAR model**	50.3374	4.0229e-09 (<0.001)
**Wald test of SDM vs SLM model**	59.3479	7.9402e-10 (<0.001)
**LR test of SDM vs SLM model**	56.6618	2.1389e-10 (<0.001)
**LM Test of fixed effect and random effect**	20.3087	0.0878

According to the test results, the SDM containing the fixed effect is more suitable for this study though the fixed effect still need to be further analyzed. Therefore, the preliminary model can be formulated as follows:

1) Spatial Durbin model: $lny_{it}=\rho Wlny_{it}+lnX*\beta_{1}+WlnX*\beta_{2}+\gamma_{i}+\delta_{t}+\epsilon_{it}$

2) Dynamic spatial Durbin model: $lny_{it}=\rho_{1}lny_{it-1}+\rho_{2}Wlny_{it}+\rho_{3}Wlny_{it-1}+lnX*\beta_{1}+WlnX*\beta_{2}+\gamma_{i}+\delta_{t}+\epsilon_{it}$

LnX is the explanatory variable matrix. $lny_{it}\left(-1\right)$ represents the first order lagged term of the explained variable. The spatial fixed effect and the temporal fixed effect are respectively reflected in $\gamma_{i}$ and $\delta_{t}$ 。

According to the comparison of the results of the three spatial Durbin models (see Table 5 for details), the time fixed effect model has the highest fitting degree. Thus, we take this model as the benchmark regression model to further develop the dynamic spatial Durbin model. Regression results shows that the coefficient in front of the explanatory variable (lnjal) is positive and significant, indicating that the improvement of the labor institutional environment can promote the spatial agglomeration of the labor force. From the perspective of the model, there is a positive spatial spillover effect of the labor agglomeration, showing the spatial distribution characteristics of “high, high, low, low”; however, the variable of the labor system environment (w * lnjal) does not show the spatial Durbin effect, while the variable of regional GDP (w * lnGDP) shows the spatial Durbin effect. The reason is related to the implementation of development strategies in major regions such as the Beijing-Tianjin-Hebei region and Yangtze River Delta in recent years, which the coordinated development of the regional economy promotes the labor agglomeration. The effects in Spatial Durbin Model (time fixed model) is shown on Table 6.

**TABLE 5:** Regression results of spatial durbin model (SDM).

	SDM			Dynamic SDM		
	Time fixed	Space fixed	Both fixed	Lagged model Ⅰ	Lagged model Ⅱ	Lagged model Ⅲ
Lny (−1)				0.97771^***^	0.98129^***^	
				−0.003		−0.003
Lnjal	0.70785^**^	0.14266^**^	0.14637^**^	0.12824^***^	1.37802^***^	0.12143^***^
	(0.345)	(0.061)	(0.064)	−0.022	−0.331	−0.022
Lnss	0.22398^**^	0.13100^***^	0.11822^***^	0.01231^*^	0.17550^*^	0.01166^*^
	(0.098)	(0.041)	(0.041)	−0.006	−0.096	−0.006
Lngdp	0.70162^***^	−0.04854	−0.03413	−0.0036	0.59681^***^	−0.00567
	(0.104)	(0.046)	(0.046)	−0.007	−0.102	−0.007
Lninvest	0.36521^***^	−0.04972^***^	−0.05801^***^	0.01003^**^	0.45219^***^	0.00839^*^
	(0.067)	(0.017)	(0.018)	−0.004	−0.065	−0.004
Lnrd	−0.12634^***^	−0.04059^***^	−0.03885^***^	−0.00061	−0.06903^**^	−0.00037
	(0.035)	(0.013)	(0.013)	−0.002	−0.033	−0.002
Lntrade	−0.05453^***^	−0.03823^***^	−0.03359^***^	0.00151^**^	−0.05995^***^	0.00171^**^
	(0.010)	(0.011)	(0.011)	−0.001	−0.01	−0.001
Wlny (−1)					1.02990^***^	−0.0069
					−0.137	−0.082
W*lnjal	1.44046^*^	−0.09700	0.04118	0.63644^***^	5.58989^***^	0.60775^***^
	(0.836)	(0.108)	(0.155)	−0.053	−0.803	−0.053
W*lnss	0.80634^***^	−0.26909^***^	−0.45983^***^	−0.02018	0.26061	−0.02159
	(0.303)	(0.078)	(0.107)	−0.02	−0.298	−0.02
W*lngdp	−0.58477^**^	0.50245^***^	0.52406^***^	0.03966^**^	−0.62706^**^	0.04194^**^
	(0.282)	(0.094)	(0.144)	−0.018	−0.278	−0.018
W*lninvest	−0.27599	−0.10082^***^	−0.14190^***^	0.00117	−0.42660^**^	0.00234
	(0.174)	(0.036)	(0.045)	−0.011	−0.169	−0.011
W*lnrd	−0.27530^**^	0.07853^***^	0.06223^**^	−0.00992	−0.17842^*^	−0.00919
	(0.108)	(0.022)	(0.030)	−0.007	−0.107	−0.007
W*lntrade	0.07139^***^	−0.00516	−0.01228	0.00405^**^	0.07134^***^	0.00380^**^
	(0.025)	(0.019)	(0.027)	−0.002	−0.024	−0.002
W*lny	0.58903^***^	0.42363^***^	0.30203^***^	0.01933^***^	0.02124	0.01556
	(0.055)	(0.067)	(0.074)	−0.006	−0.115	−0.083
$\sigma^{2}$	0.10915^***^	0.00365^***^	0.00347^***^	0.00045^***^	0.10302^***^	0.00044^***^
	(0.007)	(0.000)	(0.000)	0	−0.006	0
N	510	510	510	480	480	480
r2	0.19248	0.00056	0.06180	0.9976	0.74969	0.99776
r2_w	0.58086	0.67425	0.53411	0.93467	0.43308	0.93737

(*means *p* < 0.1, ** means *p* < 0.05,*** means *p* < 0.01 respectively).

**TABLE 6:** the effects in SDM(time fixed model).

Lny	Coef	Std. Err	Z	*p*
Direct effect				
lnjal	0.952,989	0.398,224	2.39	0.017
lnss	0.350,688	0.124,058	2.83	0.005
lngdp	0.68211	0.100,067	6.82	0.000
lninvest	0.353,949	0.07581	4.67	0.000
lnrd	−0.17351	0.033867	−5.12	0.000
lntrade	−0.04955	0.010446	−4.74	0.000
Indirect effect				
lnjal	4.385,398	2.111,649	2.08	0.038
lnss	2.177,519	0.811,577	2.68	0.007
lngdp	−0.38087	0.61231	−0.62	0.534
lninvest	−0.14423	0.412,995	−0.35	0.727
lnrd	−0.82074	0.267,116	−3.07	0.002
lntrade	0.090154	0.057952	1.56	0.12
Total effect				
lnjal	5.338,387	2.371,665	2.25	0.024
lnss	2.528,207	0.901,483	2.8	0.005
lngdp	0.301,241	0.637,217	0.47	0.636
lninvest	0.209,722	0.458,282	0.46	0.647
lnrd	−0.99425	0.281,637	−3.53	0.000
lntrade	0.040606	0.062449	0.65	0.516

As for the dynamic spatial Durbin model, both the lagged term (time-lagged term) of the explanatory variable and the lagged term (spatial lagged term) of the spatial explanatory variable have lagged effects. The coefficient of the core explanatory variable in the lagged model I becomes smaller after adding the lagged term. Specifically, the effect of the core explanatory variable on the explanatory variable, can be divided into the short-term effect and long-term effect. Both coefficients of the short-term effect and the long-term effect are positive, which indicates that a good labor market institution environment has a long-term cumulative effect. In other words, the long-term maintenance of a good labor institution environment can stimulate the further agglomeration of the labor force. Meanwhile, the coefficient of the spatial lagged term in lagged model II is positive, and the spatial lagged term also has a positive long-term external spillover effect. In the long run, the good institutional environment in a region is good for economic development and will bring about labor agglomeration, which will have a spatial spillover effect and have a positive impact on labor agglomeration in adjacent regions. So, the coefficient of the spatial lagged term in lagged model II is positive. Investment in the regression model refers to the whole investment in the society, including infrastructure investment and real estate investment. These sectors have huge labor demand. Therefore, when there is more investment in this region, it will have a negative impact on labor agglomeration in adjacent regions.

The coefficient of nearest neighbor space weight was used for the robustness test. The results are almost consistent with the above conclusions, indicating that the above conclusions are reliable. The R&D level in this model has a negative effect on the total labor agglomeration, but the coefficient in another model is positive when the explanatory variable is the total labor force with a university degree or above. It is concluded that the technological progress and the agglomeration of professional and technical personnel are synchronized. This paper also extend the regression by changing the explained variable, table a_1 in the appendix is regression result; by changing the weight matrices, table a_2 in the appendix is regression result.

## Conclusion and the Direction for Further Study

The spatial agglomeration of the labor force in China is the trend of economic and social development, and its characteristics and paths have changed. The rural surplus labor is almost exhausted and the trend of labor agglomeration shifts to the transfer between cities instead of the migration from rural to urban areas. The reason for labor migration changes from direct economic motivation to comprehensive factors (direct economic motivation and indirect economic motivation). During this process, the institutional environment related to employment plays an important role in the labor agglomeration. Based on the empirical analysis of provincial panel data from 2001 to 2017, the following conclusions are drawn: 1) Establishing a good social contract relationship with workers and providing good public services can promote labor agglomeration; 2) Properly dealing with labor disputes, building harmonious labor relations and improving the labor market institutions environment can facilitate the labor agglomeration; 3)Enhancing the ability of scientific and technological innovation in a region can stimulate the labor agglomeration and the gathering of technical personnel.

The directions of further study include, 1) How to measure the labor market institutional environment more accurately can be studied in depth. This paper uses a single index of the rate of labor dispute settlement, which can not fully and accurately represent the labor market institutional environment of a region, and there are requirements for improvement. 2) The mechanism of spatial correlation of labor market institutional environment needs to be further analyzed in theory.

① Source: China floating population development report 2018, social science literature press: Beijing, 2018.

② It should be noted that: 1) the data of the Pearl River Delta is the statistical data of Guangdong Province, the data of the Yangtze River Delta is the sum of Shanghai, Zhejiang and Jiangsu provinces, the central region refers to Shanxi, Jiangxi, Henan, Hubei, Hunan and Anhui provinces, and the western region refers to Inner Mongolia, Guangxi, Chongqing, Sichuan, Guizhou, Yunnan, Tibet, Shaanxi, Gansu, Qinghai, Ningxia and Xinjiang and other provinces and cities; 2) before 2010, the total employment of each region was taken out of the total employment of the whole country; after 2010, there was no statistics on the total employment of each region, so it was replaced by the proportion of urban employment of each region in the employment of the whole country; 3) the data of 2006 is not available, which was smoothed by the average of 2005 and 2007.

## Data Availability

The original contribution presented in the study are included in the article/Supplementary Material, further inquiries can be directed to the corresponding author.
